# Managing laboratory automation: integration and informatics in drug discovery

**DOI:** 10.1155/S1463924600000298

**Published:** 2000

**Authors:** Charles J. Manly

**Affiliations:** Discovery Technologies Neurogen Corporation Branford CT USA

## Abstract

Drug discovery today requires the focused use of laboratory automation and other resources in combinatorial chemistry and high-throughput screening (HTS). The ultimate value of both combinatorial chemistry and HTS technologies and the lasting impact they will have on the drug discovery process is a chapter that remains to be written. Central to their success and impact is how well they are integrated with each other and with the rest of the drug discovery processes-informatics is key to this success. This presentation focuses on informatics and the integration of the disciplines of combinatorial chemistry and HTS in modern drug discovery. Examples from experiences at Neurogen from the last five years are described.


Journal of Automated Methods & Management in Chemistry, Vol. 22, No. 6 (November–December 2000) pp. 169–170

### Managing laboratory automation: integration and informatics in drug discovery

**Charles J. Manly**

*Vice President, Discovery Technologies, Neurogen Corporation, Branford, CT, USA*

*Drug discovery today requires the focused use of laboratory automation and other resources in combinatorial chemistry and high-throughput screening (HTS). The ultimate value of both combinatorial chemistry and HTS technologies and the lasting impact they will have on the drug discovery process is a chapter that remains to be written. Central to their success and impact is how well they are integrated with each other and with the rest of the drug discovery processes—informatics is key to this success. This presentation focuses on informatics and the integration of the disciplines of combinatorial chemistry and HTS in modern drug discovery. Examples from experiences at Neurogen from the last five years are described.*

### Introduction

Neurogen Corporation is a pharmaceutical company focusing on central nervous system (CNS) disorders. Several years ago, we began to develop methodology, now named 'AIDD$^{SM}$' for 'Accelerated Intelligent Drug Discovery', with the aim of streamlining and optimizing:

- the generation of lead series,
- the exploration and characterization of lead series,
- the optimization of leads, and
- the optimization of clinical development candidates.

AIDD$^{SM}$ accomplishes this through tight integration (via intranet deployed informatics) of combinatorial chemistry, high-throughput pharmacology and computational chemistry. AIDD$^{SM}$ itself is tightly integrated with the drug discovery effort and especially with medicinal chemistry itself.

The focus of AIDD$^{SM}$ is the ability to greatly enhance the drug discovery cycle of synthesis, data generation, data analysis and modelling, and to prioritize both synthesis and screening—thus completing the cycle—on thousands of compounds every two weeks (figure 1). Additionally, this is accomplished with the following.

- Very small staff resources (20–25 full-time employees).
- Ability to synthesize 400,000 samples per year (as either mixtures or individual samples) with purification and quality assessment.
- Biological data generation of 300 000 samples per month.
- Cycle time of two weeks
- Targeted efficiency gains through computational chemistry and data-mining of 10 × to well over 50 × over random.
- Ability to prosecute 13–15 programs simultaneously in the above manner.

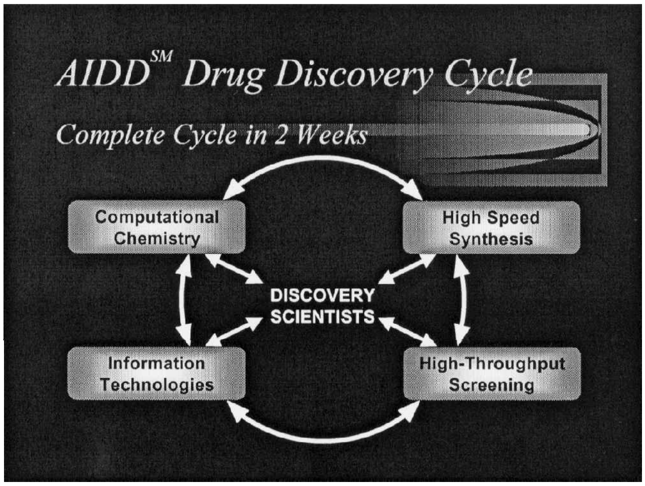

*Figure 1. AIDD$^{SM}$ drug discovery cycle.*

### Virtual library

The AIDD$^{SM}$ virtual library is managed by Neurogen's ISLANDS$^{SM}$ technology and is a representation of all compounds that can be made from the existing reactive fragment database and synthesis protocol database. Thus this virtual library is a very specific and dynamic set of compounds that can easily be millions or billions of molecules in size. The ISLANDS$^{SM}$ technology managing the virtual library is key to AIDD$^{SM}$ virtual screening processes as well as to workflow operations. The ISLANDS$^{SM}$ software makes it possible to define and register 50 000 compounds from the virtual library easily and quickly (in 10 minutes). After definition and registration, not only do the compounds exist electronically in databases for use in AIDD$^{SM}$ but also ISLANDS$^{SM}$ has generated all the information required in the synthesis itself. The reagents required, the synthesis, reaction workup, and quality control protocols to be used by the synthesis robotics, and all tracking information (sample number, plate number, well locations) have been automatically generated and specified with no further input from the user required.

### Virtual screening

A key concept of AIDD$^{SM}$ is the effective prioritization of both synthesis and screening resources through virtual screening. Proprietary, unattended and continuous molecular modelling and data-mining strategies termed 'On-Line Continuous Modelling' (OLCM) provide models for virtual screening of both the virtual library and the archive of actual compounds. These models work in

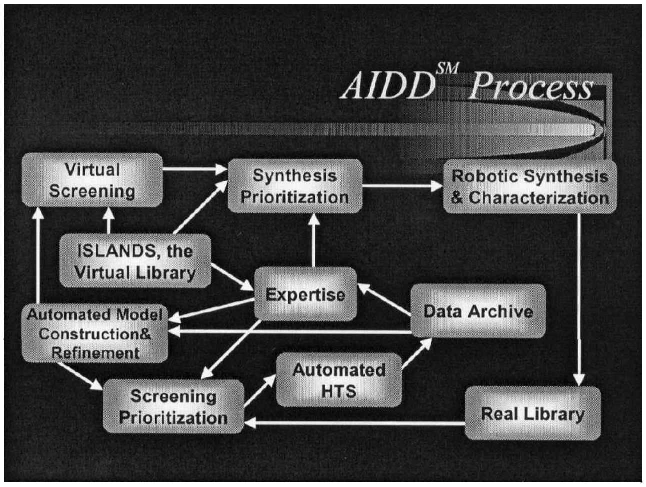

*Figure 2. The AIDD$^{SM}$ process.*

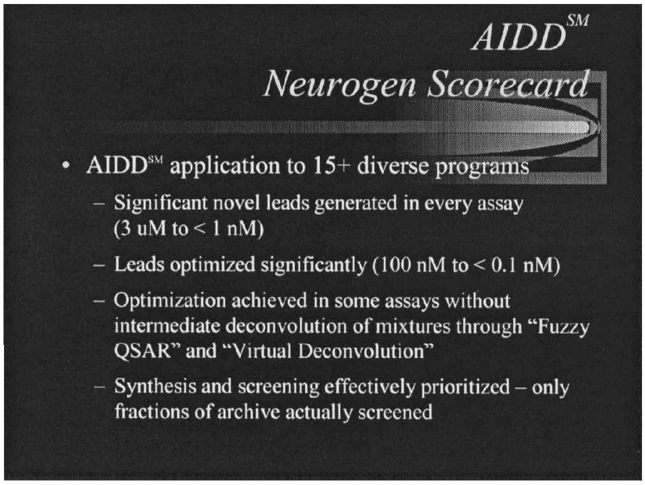

*Figure 3. AIDD$^{SM}$ scorecard.*

concert with ISLANDS$^{SM}$ for virtual screening of the virtual library (figure 2).

### On-line continuous modelling

From the inception of our work on AIDD$^{SM}$, we planned to perform computational chemistry modelling with a novel portfolio approach. A portfolio of modelling strategies could be expected to provide useful models in a variety of cases when no one strategy could be expected to perform well in every situation. Compare this to a stock portfolio where the expectation is that the portfolio will increase in value with time even though this cannot be expected of any one particular stock. The AIDD$^{SM}$ portfolio of OLCM strategies includes a variety of chemical descriptor types and a variety of modeling methods. Fuzzy methods and machine methods have been very effective. Both artificial neural networks and recursive partitioning methodologies are also used routinely in AIDD$^{SM}$ OLCM studies.

A core principle of AIDD$^{SM}$ and OLCM is the prediction, prioritization and targeting of populations of compounds instead of individual compounds. This makes it possible to routinely achieve significant benefits, by increasing the probability of activity in each two-week cycle. Efficiency gains or targeting enhancements seen in AIDD$^{SM}$ from this approach are routinely $10\times$ to more than $50\times$ increased.

### Results

AIDD$^{SM}$ has been applied to over 15 diverse programs at Neurogen: in each program AIDD$^{SM}$ resulted in novel leads that were readily optimized to significant levels of activity (figure 3).

Neurogen has been applying AIDD$^{SM}$ technology to the optimization of drug-like properties within projects toward the generation of development candidates. These efforts have resulted in more efficient optimization of candidate ADME and PK properties such as metabolic half-life, cytochrome P450 activity, and others.

### Summary

An overview of the AIDD$^{SM}$ Drug Discovery System at Neurogen has been given. Specific examples from active project areas were presented. The importance of integration of disciplines and of pragmatism in balancing the individual components of drug discovery was stressed.

